# Electrochromic Devices Based on Poly(2,6-di(9H-carbazol-9-yl)pyridine)-Type Polymer Films and PEDOT-PSS

**DOI:** 10.3390/polym10060604

**Published:** 2018-05-31

**Authors:** Chung-Wen Kuo, Bo-Wei Wu, Jeng-Kuei Chang, Jui-Cheng Chang, Li-Ting Lee, Tzi-Yi Wu, Tsung-Han Ho

**Affiliations:** 1 Department of Chemical and Materials Engineering, National Kaohsiung University of Science and Technology, Kaohsiung 80778, Taiwan; welly@cc.kuas.edu.tw (C.-W.K.); 1104311140@gm.kuas.edu.tw (B.-W.W.); thho@cc.kuas.edu.tw (T.-H.H.); 2 Institute of Materials Science and Engineering, National Central University, Taoyuan 32001, Taiwan; jkchang@ncu.edu.tw; 3 Department of Chemical Engineering and Materials Engineering, National Yunlin University of Science and Technology, Yunlin 64002, Taiwan; d700215@gmail.com; 4 Department of Materials Science and Engineering, Feng Chia University, Taichung 40724, Taiwan; ltlee@fcu.edu.tw

**Keywords:** electrochromic polymer, spectroelectrochemistry, electrochemical polymerization, optical contrast, poly(3,4-ethylenedioxythiophene)-poly(styrene sulfonic acid)

## Abstract

2,6-Di(9H-carbazol-9-yl)pyridine (DiCP) was synthesized and its corresponding homopolymer (PDiCP) and copolymers (P(DiCP-*co*-CPDT), P(DiCP-*co*-CPDT2), P(DiCP-*co*-CPDTK), and P(DiCP-*co*-CPDTK2)) were synthesized electrochemically. The anodic copolymer with DiCP:cyclopentadithiophene ketone (CPDTK) = 1:1 feed molar ratio showed high transmittance change (Δ*T*%) and colouration efficiency (*η*), which were measured as 39.5% and 184.1 cm^2^ C^−1^ at 1037 nm, respectively. Electrochromic devices (ECDs) were composed of PDiCP, P(DiCP-*co*-CPDT), P(DiCP-*co*-CPDT2), P(DiCP-*co*-CPDTK), and P(DiCP-*co*-CPDTK2) as anodically-colouring polymers, and poly(3,4-ethylenedioxythiophene)-poly(styrene sulfonic acid) (PEDOT-PSS) as cathodically-colouring polymers. P(DiCP-*co*-CPDTK)/PEDOT-PSS ECD showed light silverish-yellow at 0.0 V, light grey at 0.7 V, grey at 1.3 V, light greyish blue at 1.7 V, and greyish blue at 2.0 V. Moreover, P(DiCP-*co*-CPDTK)/PEDOT-PSS ECD presented high Δ*T* (38.2%) and high *η* (633.8 cm^2^ C^−1^) at 635 nm.

## 1. Introduction

Organic electroactive materials (OEM) have played a role in science and commercial electronic devices due to their benefits of facile processing, flexibility of molecular design and synthesis, and reversibility between oxidation and reduction processes [1]. Conjugated polymers (CPs) were widely-studied organic electroactive materials due to their potential applications in supercapacitors [2], light-emitting diodes [3,4,5], electrochromic devices (ECDs) [6,7,8], solar cells [9,10], fluorescent sensors [11,12], catalysts for methanol electroxidation [13,14,15], and organic transistors [16]. Among these applications, scientists have focused immensely in ECDs by virtue of their multicolour and energy-saving characteristics after applying a voltage [17].

During the last decade, several CPs, such as polythiophenes (PTh) [18], polycarbazoles (PCz) [19,20], polytriphenylamines [21,22], polyanilines (PANI) [23], polyindoles [24,25], polyazulenes [26], and polyimides [27] have been widely studied for use as electrochromic materials. Among these CPs, PCz showed a light colour in the neutral state and a deep colour in oxidized state. Carbazole derivatives can be functionalized at the 9-, 3,6-, and 2,7-positions and a wide variation of alkyl and phenyl chains can be incorporated at the 9-, 3,6-, and 2,7-positions of carbazole derivatives. Metin et al. synthesized a star-shaped compound (2,4,6-tris((9H-carbazol-2-yl)oxy)-1,3,5-triazine (CTR)) and reported that P(CTR) switches between dark turquoise and transparent with an optical contrast of 50%, the ∆*T* of P(CTR)/P(EDOT) ECD was measured as 32% at 615 nm [28]. Zhang et al. reported that a multichromic copolymer (P(CDPN-*co*-EDOT)) revealed claret red, green, cadet blue, and blue from neutral to oxidized states. P(CDPN-*co*-EDOT) film showed ∆*T* of 36% in the visible region and a ∆*T* of 43% in the near-infrared region [29]. PTh and their derivatives were usually employed to diminish the band gap and improve the conjugation of the polymer backbone. The derivatives of PTh (poly(3,4-ethylenedioxythiophene)s (PEDOT) and poly(3,4-(2,2-dimethylpropylenedioxy)thiophene)s (PProDOT-Me_2_)) comprised two electron-donating oxygen atoms at 3,4-positions of thiophene ring, which diminished the onset potentials of oxidation for polymer films and diminished the band gaps of polymer films. Moreover, PEDOT and PProDOT-Me_2_ showed high transmissivity in the doped state and low transmissivity in the undoped state, which made PEDOT and PProDOT-Me_2_ promising candidates for use as cathodic electrodes in electrochromic devices [30]. Furthermore, copolymerization is an efficient and promising way to improve electrochromic properties of the CPs. Copolymerization of dissimilar monomers containing numerous distinct units can bring about interesting combinations of the electrochromic characteristics. For this affair, copolymers based on carbazole and bithiophene derivatives are copolymerized in the study. 2,6-di(9H-carbazol-9-yl)pyridine comprises two carbazole groups linked by a pyridine ring, the carbazole and pyridine groups are electron-donating and electron-withdrawing groups, respectively. Cyclopentadithiophene ketone (CPDTK) comprises two thiophene rings linked by a single bond and a carbonyl group at the 2-position and 3-position of thiophene rings, respectively, the thiophene and carbonyl groups are electron-donating and electron-withdrawing groups, respectively. Electrochromic polymers based on monomers possessing donor-acceptor-donor (D-A-D) units can adjust the colour in their doped and dedoped states and the colour of dual type electrochromic devices at various voltages.

Up to now, the incorporation of a 2,6-di(9H-carbazol-9-yl)pyridine unit into electrochromic polymer backbones and the electrochromic behaviors of poly(2,6-di(9H-carbazol-9-yl)pyridine)s at various potentials have not been reported. In the present work, 2,6-di(9H-carbazol-9-yl)pyridine (DiCP) is synthesized and its corresponding homopolymer (PDiCP) and copolymers (P(DiCP-*co*-CPDT), P(DiCP-*co*-CPDT2), P(DiCP-*co*-CPDTK), and P(DiCP-*co*-CPDTK2)) are synthesized using electrochemical copolymerization. The spectroelectrochemical behaviours of PDiCP, P(DiCP-*co*-CPDT), P(DiCP-*co*-CPDT2), P(DiCP-*co*-CPDTK), and P(DiCP-*co*-CPDTK2) films at various potentials are comprehensively studied. It is interesting to explore that the slight structural modifications of the bithiophene derivatives give rise to diverse spectroelectrochemical behaviours. Moreover, five ECDs were fabricated using PDiCP, P(DiCP-*co*-CPDT), P(DiCP-*co*-CPDT2), P(DiCP-*co*-CPDTK), and P(DiCP-*co*-CPDTK2) films as the anodically-colouring polymers, poly(3,4-ethylenedioxythiophene)-poly(styrene sulfonic acid) (PEDOT-PSS) as the cathodically-colouring polymer. The optical contrast, colouration efficiency, switching property, optical memory, and redox stability of the five ECDs were also studied.

## 2. Materials and Methods

### 2.1. Materials

2,6-dibromopyridine, carbazole, and PEDOT-PSS (1.3 wt % dispersion in water) were purchased from Aldrich (St. Louis, MO, USA). 4H-cyclopenta[2,1-b:3,4-b’]dithiophene (CPDT) and cyclopentadithiophene ketone were purchased from Tokyo Chemical (Tokyo, Japan), PMMA (*M*_w_ = 350,000), LiClO_4_, and propylene carbonate were purchased from Acros organics (Geel, Belgium), Aldrich (St. Louis, MO, USA), and Alfa Aesar (Haverhill, MA, USA), respectively, and were used as received.

### 2.2. Synthesis of 2,6-di(9H-carbazol-9-yl)pyridine (DiCP)

2,6-dibromopyridine (3.55 g, 15 mmol), carbazole (10.03 g, 60 mmol), K_2_CO_3_ (8.29 g, 60 mmol), Cu powder (2.41 g, 38 mmol), and 30 mL triethylene glycol dimethyl ether were added in a two-neck round-bottom flask and stirred at 175 °C for 48 h. Triethylene glycol dimethyl ether was vaporized using a rotavapor under reduced pressure, and the crude product was purified through column chromatography (silica gel with an eluent of dichloromethane/hexane mixture). Recrystallization from toluene to give desired DiCP. Yield: 62%. ^1^H NMR (700 MHz, DMSO-*d*_6_): *δ* 8.42 (dd, 1H, pyridine-H), 8.28 (d, 4H, carbazole-H), 7.93 (d, 4H, carbazole-H), 7.88 (d, 2H, pyridine-H), 7.45 (dd, 4H, carbazole-H), 7.35 (dd, 4H, carbazole-H). Elem. Anal. Calcd. for C_29_H_19_N_3_: C, 85.06%; H, 4.68%; N, 10.26%. Found: C, 84.95%; H, 4.59%; N, 10.21%.

### 2.3. Electrochemical Polymerization of PDiCP, P(DiCP-co-CPDT), P(DiCP-co-CPDT2), P(DiCP-co-CPDTK), and P(DiCP-co-CPDTK2) Films

**Figure 1:**
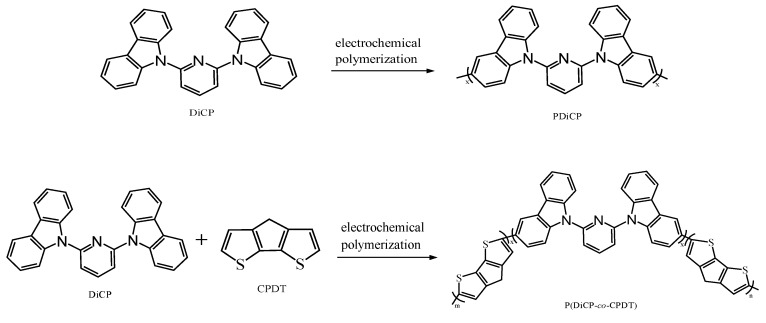

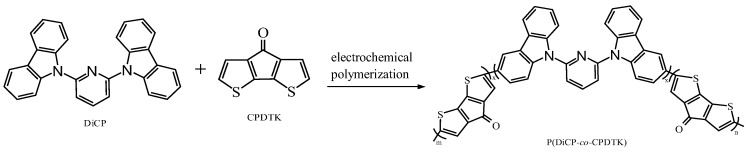
The electrochemical polymerization schemes of PDiCP, P(DiCP-*co*-CPDT), and P(DiCP-*co*-CPDTK).

PDiCP, P(DiCP-*co*-CPDT), P(DiCP-*co*-CPDT2), P(DiCP-*co*-CPDTK), and P(DiCP-*co*-CPDTK2) films were electrosynthesized using cyclic voltammetry (CV) between 0.0 and 1.5 V with a scan rate of 100 mV s^−1^ in an acetonitrile (ACN)/dichloromethane (DCM) (1:1, by volume) solution containing 0.2 M LiClO_4_ as an electrolyte, the feed species and molar ratio of anodically-colouring polymers are presented in Table 1. The counter and reference electrodes of the electrochemical system are a platinum wire and an Ag/AgCl (3 M KCl) electrode, respectively. The cathodic PEDOT-PSS film is prepared using spin-coating methods, and the spin condition for preparing PEDOT-PSS film is 2000 rpm. The active area of polymer electrode is 1.0 cm × 1.5 cm. The electrochemical polymerization schemes of PDiCP, P(DiCP-*co*-CPDT), and P(DiCP-*co*-CPDTK) are shown in Figure 1.

### 2.4. Preparation of Dual-Type Electrochromic Devices

The electrolyte consisting of PMMA, LiClO_4_, PC, and ACN was prepared according to previously published work [31], and the electrolyte was coated on anodic PDiCP, P(DiCP-*co*-CPDT), P(DiCP-*co*-CPDT2), P(DiCP-*co*-CPDTK), and P(DiCP-*co*-CPDTK2) films. Eventually, the cathodic PEDOT-PSS film was placed onto the electrolyte to fabricate electrochromic devices.

### 2.5. Electrochemical and Electrochromic Characterization

The electrochemical characterizations of polymer films were carried out in three-component cells. An ITO coated glass, Pt wire, and Ag/AgCl (3 M KCl) electrode were used as working, counter, and reference electrodes, respectively. The electrochromic experiments and double potential chronoamperometry were implemented with a Hitachi spectrophotometer and a CHI627D (CH Instruments, Austin, TX, USA) electrochemical analyser.

## 3. Results and Discussion

### 3.1. Electrochemical Polymerizations

**Figure 2:**
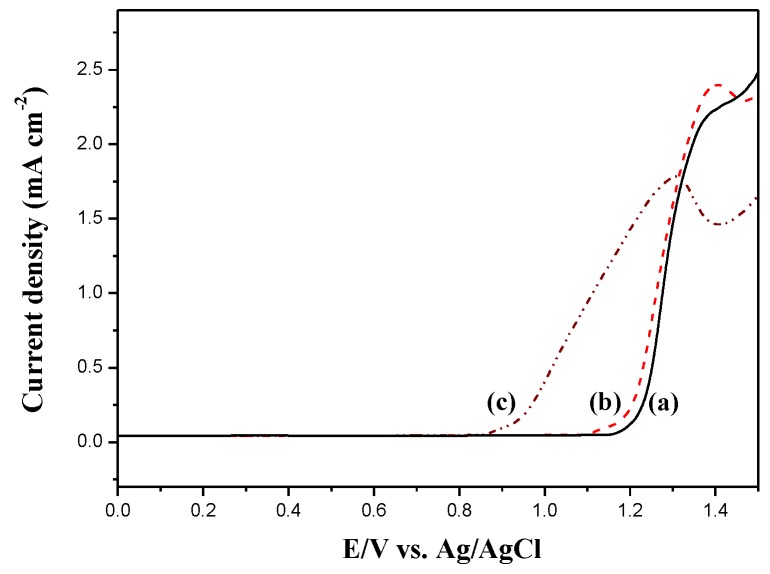
Electrooxidation of (**a**) 4 mM DiCP; (**b**) 4 mM CPDTK; and (**c**) 4 mM CPDT in 0.2 M LiClO_4_ containing ACN/DCM (1:1, by volume) solution at a scan rate of 100 mV s^−1^.

**Figure 3:**
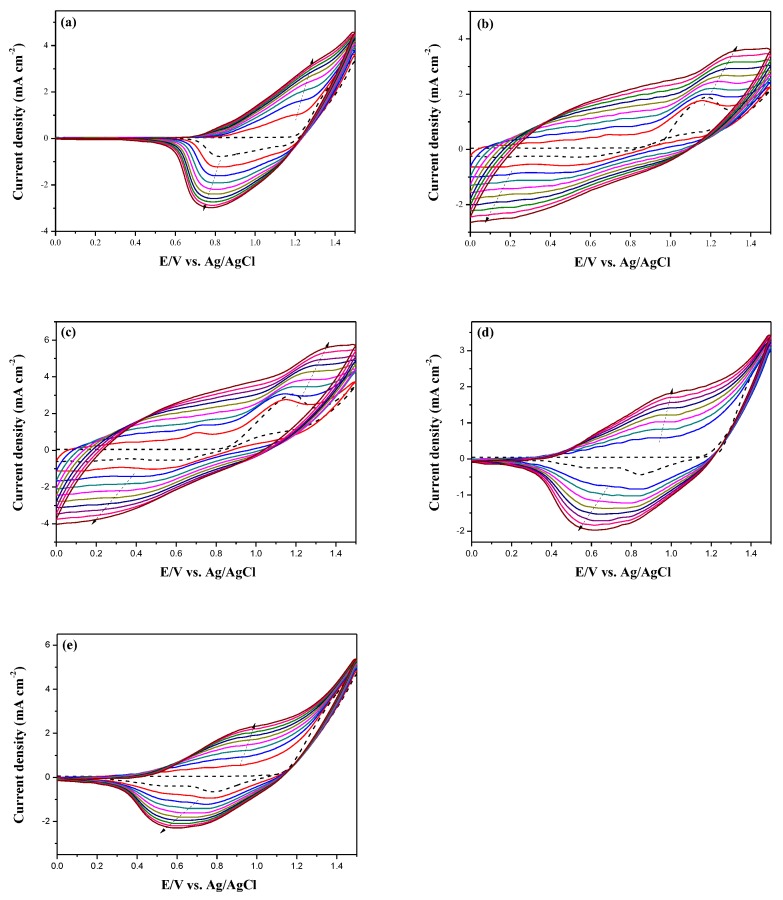
Electrochemical synthesis of (**a**) PDiCP; (**b**) P(DiCP-*co*-CPDT); (**c**) P(DiCP-*co*-CPDT2); (**d**) P(DiCP-*co*-CPDTK); and (**e**) P(DiCP-*co*-CPDTK2) in ACN/DCM (1:1, by volume) solution at 100 mV s^−1^ on the ITO working electrode.

The electrooxidation of DiCP, CPDTK, and CPDT in 0.2 M LiClO_4_ ACN/DCM solution were shown in Figure 2. The onset potential of oxidation for DiCP, CPDT, and CPDTK were 1.15, 0.95, and 1.10 V, respectively. The onset potential of CPDT is smaller than that of CPDTK, this can be attributed to the withdrawing keto groups of CPDTK increasing the onset potential significantly. Moreover, the differences between onset potential of oxidation for DiCP vs. CPDTK and DiCP vs. CPDT are less than 0.2 V, indicating the copolymerizations of DiCP with CPDTK (or CPDT) are practicable. Figure 3 shows the electrochemical synthesis of PDiCP, P(DiCP-*co*-CPDT), P(DiCP-*co*-CPDT2), P(DiCP-*co*-CPDTK), and P(DiCP-*co*-CPDTK2) in 0.2 M LiClO_4_/ACN/DCM solution (ACN:DCM = 1:1, by volume) at 100 mV s^−1^ on ITO working electrode.

The current densities of the redox peaks increase with increasing scanning CV cycles, implying both deposition of the polymer films and that the deposited polymers are conducting [32]. The oxidation peaks of PDiCP, P(DiCP-*co*-CPDT), P(DiCP-*co*-CPDT2), P(DiCP-*co*-CPDTK), and P(DiCP-*co*-CPDTK2) located at 1.15, 1.10, 1.10, 0.9 and 0.9 V, respectively, whereas the reduction peaks of PDiCP, P(DiCP-*co*-CPDT), P(DiCP-*co*-CPDT2), P(DiCP-*co*-CPDTK), and P(DiCP-*co*-CPDTK2) located at 0.75, 0.45, 0.5, 0.6, and 0.6 V, respectively. The redox peaks and CV curves of P(DiCP-*co*-CPDT), P(DiCP-*co*-CPDT2), P(DiCP-*co*-CPDTK), and P(DiCP-*co*-CPDTK2) are different from the oxidation and reduction peaks and CV curves of PDiCP, indicating the formation of P(DiCP-*co*-CPDT), P(DiCP-*co*-CPDT2), P(DiCP-*co*-CPDTK), and P(DiCP-*co*-CPDTK2) films on ITO electrodes. Specific capacitances of PDiCP, P(DiCP-*co*-CPDT), P(DiCP-*co*-CPDT2), P(DiCP-*co*-CPDTK), and P(DiCP-*co*-CPDTK2) electrodes are 76, 92, 94, 85, and 88 F/g, respectively.

**Figure 4:**
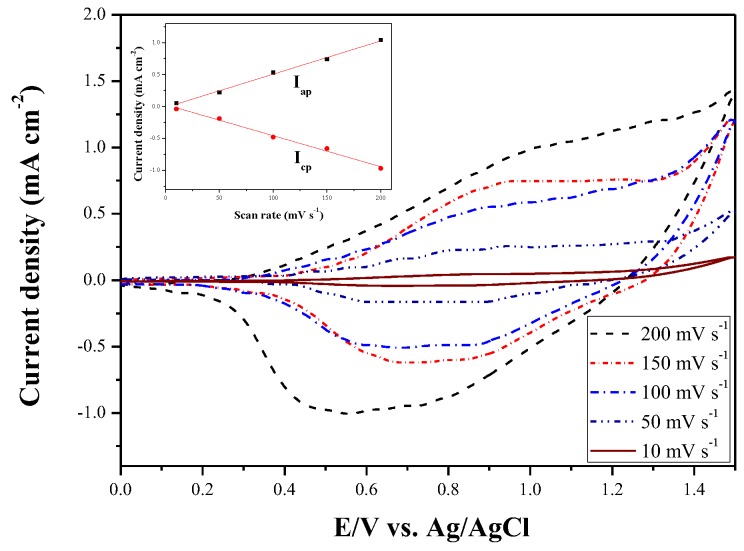
CV curves of the P(DiCP-*co*-CPDTK) films at different scan rates between 10 and 200 mV s^−1^ in 0.2 M LiClO_4_/ACN/DCM solution. Scan rate dependence of the anodic and cathodic peak current densities for P(DiCP-*co*-CPDTK) films (inset).

Figure 4 shows the CV curves of deposited P(DiCP-*co*-CPDTK) film at 10, 50, 100, 150, and 200 mV s^−1^ in 0.2 M LiClO_4_/ACN/DCM solution, the P(DiCP-*co*-CPDTK) film shows well-defined redox peaks at various scan rates and the anodic and cathodic peak current densities are proportional to the scan rates (inset of Figure 4), inferring the P(DiCP-*co*-CPDTK) film was stuck onto ITO glass and the redox process of the copolymer film was not diffusional control [33].

### 3.2. Spectroelectrochemical Characterizations of Homopolymer and Copolymer Films

**Figure 5:**
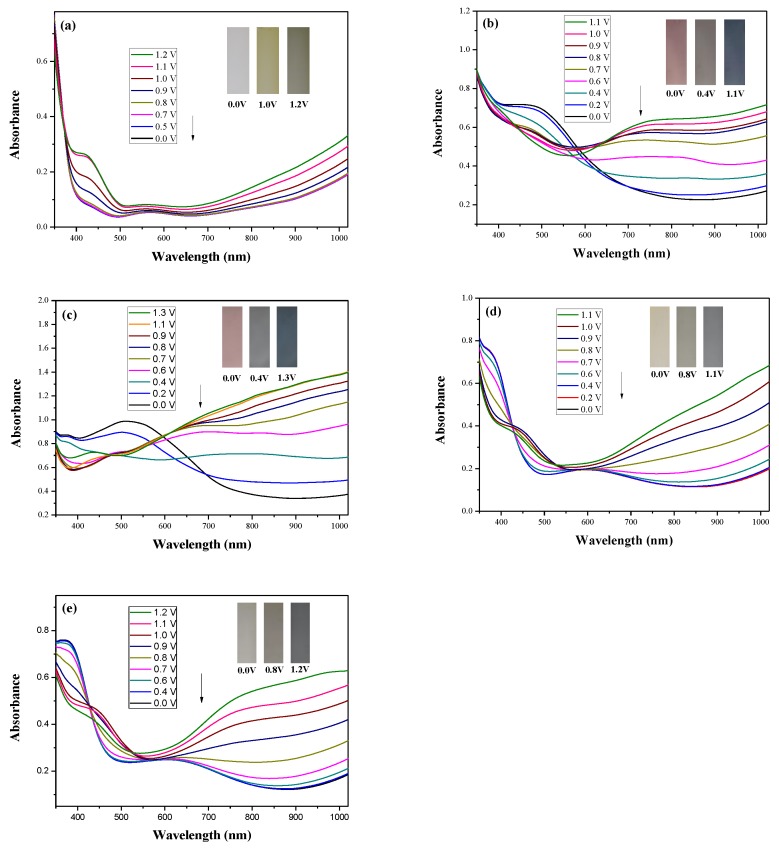
UV–VIS spectra of (**a**) PDiCP; (**b**) P(DiCP-*co*-CPDT); (**c**) P(DiCP-*co*-CPDT2); (**d**) P(DiCP-*co*-CPDTK); and (**e**) P(DiCP-*co*-CPDTK2) electrodes on ITO in 0.2 M LiClO_4_ containing ACN/DCM (1:1, by volume) solution.

Figure 5 shows the spectroelectrochemical spectra of PDiCP, P(DiCP-*co*-CPDT), P(DiCP-*co*-CPDT2), P(DiCP-*co*-CPDTK), and P(DiCP-*co*-CPDTK2) films at various potentials in 0.2 M LiClO_4_/ACN/DCM solution, and that there is no prominent absorption peak of PDiCP film at 0.0 V.

Upon increasing the potential progressively, new bands appeared at 425 and 1000 nm, which could be assigned to the formation of charge carriers for PDiCP film [34]. However, P(DiCP-*co*-CPDT), P(DiCP-*co*-CPDT2), P(DiCP-*co*-CPDTK), and P(DiCP-*co*-CPDTK2) films showed π-π* (or n-π*) transition peaks at 485, 500, 392, and 395 nm, respectively, which were different to the absorption spectra of PDiCP film in the neutral state. The UV–VIS spectra of P(DiCP-*co*-CPDT) film recorded for various potentials exhibit isosbestic points, suggesting the presence of neutral and oxidized P(DiCP-*co*-CPDT) films which are active towards different irradiations at different potentials. The charge carrier bands of P(DiCP-*co*-CPDT), P(DiCP-*co*-CPDT2), P(DiCP-*co*-CPDTK), and P(DiCP-*co*-CPDTK2) films in 0.2 M LiClO_4_/ACN/DCM solution showed significant variations at 1000 nm from neutral to oxidation states.

The PDiCP film showed three kinds of colour variations from neutral to oxidation state, PDiCP film was light gray at 0.0 V, dark khaki at 1.0 V, and grey black at 1.2 V. For the copolymer films, P(DiCP-*co*-CPDT) and P(DiCP-*co*-CPDT2) films were light brown at 0.0 V, light cadet blue at 0.4 V, and navy blue at 1.1 (or 1.3 V), whereas P(DiCP-*co*-CPDTK) and P(DiCP-*co*-CPDTK2) films were light yellow at 0.0 V, grey at 0.8 V, and rock grey at 1.1 V. The colorimetric values (L*, a*, and b*) and CIE chromaticity diagrams of PDiCP, P(DiCP-*co*-CPDT2), and P(DiCP-*co*-CPDTK) films at various potentials are displayed in Table 2.

The optical band gap (*E*_g_) of PDiCP homopolymer film calculated using Planck equation was 2.58 eV:

*E*_g_ = 1241/*λ*_onset_ (1)

where the onset UV absorption wavelength (*λ*_onset_) of the π-π* transition band of PDiCP was 481 nm [35,36]. The onset oxidation potential vs. Ag/AgCl (3 M KCl) was 0.82 V, the *E*_FOC_ of ferrocene/ferrocenium vs. Ag/AgCl (3 M KCl) determined using cyclic voltammetry was 0.80 V, and the onset oxidation potential vs. *E*_FOC_ was found to be 0.02 V. The HOMO and LUMO energy levels of PDiCP determined from the onset oxidation potential with respect to the energy level of ferrocene/ferrocenium couple (−4.8 eV less than vacuum energy level) [37,38] and *E*_g_ were taken as −4.82 and −2.24 eV, respectively.

**Figure 6:**
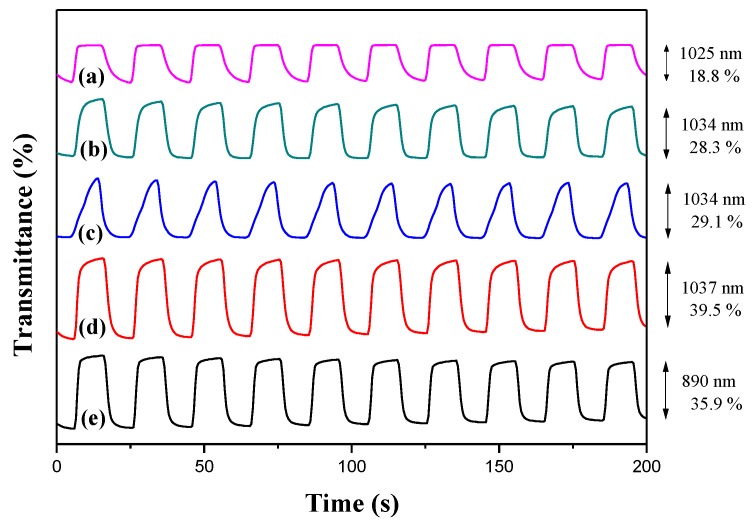
Transmittance changes of (**a**) PDiCP; (**b**) P(DiCP-*co*-CPDT); (**c**) P(DiCP-*co*-CPDT2); (**d**) P(DiCP-*co*-CPDTK); and (**e**) P(DiCP-*co*-CPDTK2) electrodes in a 0.2 M LiClO_4_ containing ACN/DCM (1:1, by volume) solution.

Figure 6 showed the electrochromic switching profiles of PDiCP, P(DiCP-*co*-CPDT), P(DiCP-*co*-CPDT2), P(DiCP-*co*-CPDTK), and P(DiCP-*co*-CPDTK2) films in 0.2 M LiClO_4_/ACN/DCM solution, which were monitored using double potential-step chronoamperometry by repeating potentials between 0.0 and 1.2 V with a time interval of 10 s. The Δ*T* of PDiCP at 1025 nm, P(DiCP-*co*-CPDT) at 1034 nm, P(DiCP-*co*-CPDT2) at 1034 nm, P(DiCP-*co*-CPDTK) at 1037 nm, and P(DiCP-*co*-CPDTK2) at 890 nm from the bleaching state to the colouration state in 0.2 M LiClO_4_/ACN/DCM solution were calculated to be 18.8, 28.3, 29.1, 39.5, and 35.9%, respectively. Among these polymer films, P(DiCP-*co*-CPDTK) film presents the highest Δ*T*, and copolymers (P(DiCP-*co*-CPDT), P(DiCP-*co*-CPDT2), P(DiCP-*co*-CPDTK), and P(DiCP-*co*-CPDTK2)) present higher Δ*T* than that of homopolymer (PDiCP) in 0.2 M LiClO_4_/ACN/DCM solution, implying the copolymerization of DiCP with CPDT (or CPDTK) monomer results in the increase of Δ*T* in 0.2 M LiClO_4_/ACN/DCM solution. The colouration switching time (*τ*_c_) and bleaching switching time (*τ*_b_) of polymer films in 0.2 M LiClO_4_/ACN/DCM solution are listed in Table 3, the *τ*_c_ and *τ*_b_ are determined at 90% of the whole transmittance change. The *τ*_c_ and *τ*_b_ of PDiCP, P(DiCP-*co*-CPDT), P(DiCP-*co*-CPDT2), P(DiCP-*co*-CPDTK), and P(DiCP-*co*-CPDTK2) films in 0.2 M LiClO_4_/ACN/DCM solution are in the range of 1.9–6.7 s.

ΔOD is the change of optical density, which can be calculated using the equation [39]:

(2) $$\Delta \mathrm{OD}=\log\left(\frac{T_{ox}}{T_{red}}\right)$$

where *T*_ox_ and *T*_red_ refer to the transmittance (%) of oxidized and neutral states, respectively. The ΔOD of PDiCP, P(DiCP-*co*-CPDT), P(DiCP-*co*-CPDT2), P(DiCP-*co*-CPDTK), and P(DiCP-*co*-CPDTK2) films at specific wavelengths in 0.2 M LiClO_4_/ACN/DCM solution are listed in Table 3. P(DiCP-*co*-CPDT2) film shows the largest ΔOD among these polymer films in 0.2 M LiClO_4_/ACN/DCM solution.

Colouration efficiency (*η*) is an important parameter for electrochromic materials and devices, and can be calculated using the formula [40]:

(3) $$\eta =\frac{\Delta \mathrm{OD}}{Q_{d}}$$

where *Q*_d_ is the injected/ejected charge of the polymer films (or devices) per active area. Table 3 lists the *η* of homopolymer and copolymer films in 0.2 M LiClO_4_/ACN/DCM solution, the *η* of PDiCP at 1025 nm, P(DiCP-*co*-CPDT) at 1034 nm, P(DiCP-*co*-CPDT2) at 1034 nm, P(DiCP-*co*-CPDTK) at 1037 nm, and P(DiCP-*co*-CPDTK2) at 890 nm are 123.8, 149.5, 80.9, 184.1, and 111.5 cm^2^ C^−1^, respectively.

The comparisons of transmittance changes and colouration efficiencies with reported polymer films were displayed in Table 4, P(DiCP-*co*-CPDTK) film showed higher transmittance change than those reported for PETCB film at 1100 nm [41], PMCzP film at 460 nm [42], and PBCz film at 1050 nm [43]. However, P(DiCP-*co*-CPDTK) film showed a lower transmittance change than those reported for P(BCz1-*co*-Inc1) film at 787 nm [44] and P(NO_2_-3Cz) film at 710 nm [45]. On the other hand, P(DiCP-*co*-CPDTK) film revealed higher *η* than those reported for P(BCz1-*co*-Inc1) [44], P(NO_2_-3Cz) [45], and PBCz films [43].

### 3.3. Spectroelectrochemical Characterizations of Electrochromic Devices

**Figure 7:**
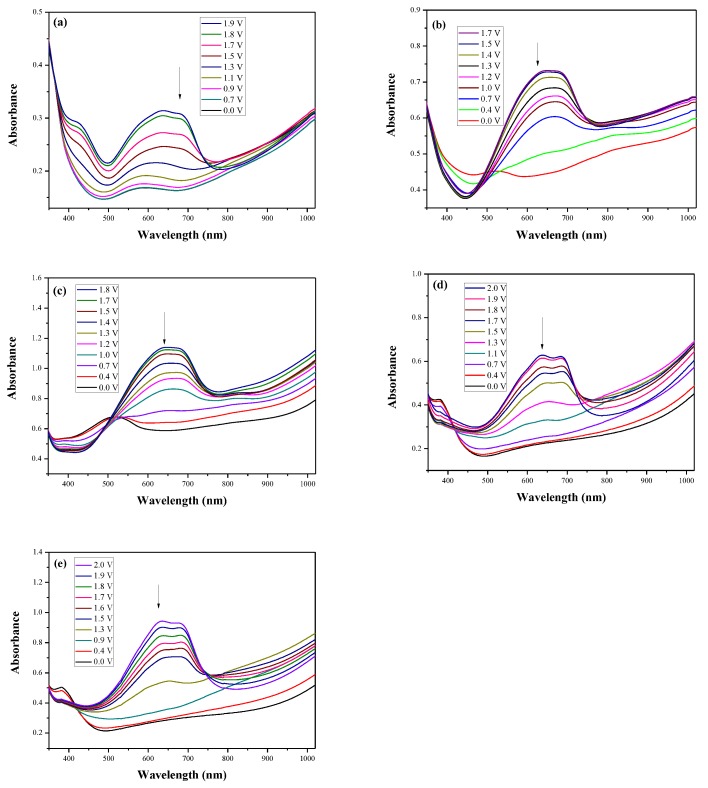
UV–VIS spectra of (**a**) PDiCP/PEDOT-PSS; (**b**) P(DiCP-*co*-CPDT)/PEDOT-PSS; (**c**) P(DiCP-*co*-CPDT2)/PEDOT-PSS; (**d**) P(DiCP-*co*-CPDTK)/PEDOT-PSS; and (**e**) P(DiCP-*co*-CPDTK2)/PEDOT-PSS ECDs.

Five dual type ECDs based on PDiCP, P(DiCP-*co*-CPDT), P(DiCP-*co*-CPDT2), P(DiCP-*co*-CPDTK), and P(DiCP-*co*-CPDTK2) films as anodically-colouring polymers and PEDOT-PSS film as cathodically-colouring polymer were fabricated, which were denominated as PDiCP/PEDOT-PSS, P(DiCP-*co*-CPDT)/PEDOT-PSS, P(DiCP-*co*-CPDT2)/PEDOT-PSS, P(DiCP-*co*-CPDTK)/PEDOT-PSS, and P(DiCP-*co*-CPDTK2)/PEDOT-PSS ECDs, respectively. Figure 7a–e displayed the UV–VIS spectra of PDiCP/PEDOT-PSS, P(DiCP-*co*-CPDT)/PEDOT-PSS, P(DiCP-*co*-CPDT2)/PEDOT-PSS, P(DiCP-*co*-CPDTK)/PEDOT-PSS, and P(DiCP-*co*-CPDTK2)/PEDOT-PSS ECDs, respectively.

At 0.0 V, PDiCP/PEDOT-PSS ECD did not show distinct absorption peaks in the ultraviolet and visible zones. At this moment, anodic PDiCP film was in its reduced state, displaying a light grey colour. The cathodic PEDOT-PSS film was in the oxidized state, displaying a limpid colour. Accordingly, the PDiCP/PEDOT-PSS ECD displayed light grey colour at 0.0 V. Upon increasing the voltages gradually, PDiCP film started to oxidize and PEDOT-PSS film began to reduce. Accordingly, new peaks at 420 and 637 nm emerged gradually and the PDiCP/PEDOT-PSS ECD presented as dark blue at 1.9 V. The main electrochromic response of PDiCP/PEDOT-PSS ECD comes from the PEDOT-PSS layer. Table 5 summarized the electrochromic photographs, colorimetric values (L*, a*, and b*) and CIE chromaticity diagrams of the P(DiCP-*co*-CPDTK)/PEDOT-PSS ECD at various potentials. P(DiCP-*co*-CPDTK)/PEDOT-PSS ECD showed light silverish-yellow at 0.0 V, light grey at 0.7 V, grey at 1.3 V, light greyish blue at 1.7 V, and greyish blue at 2.0 V.

**Figure 8:**
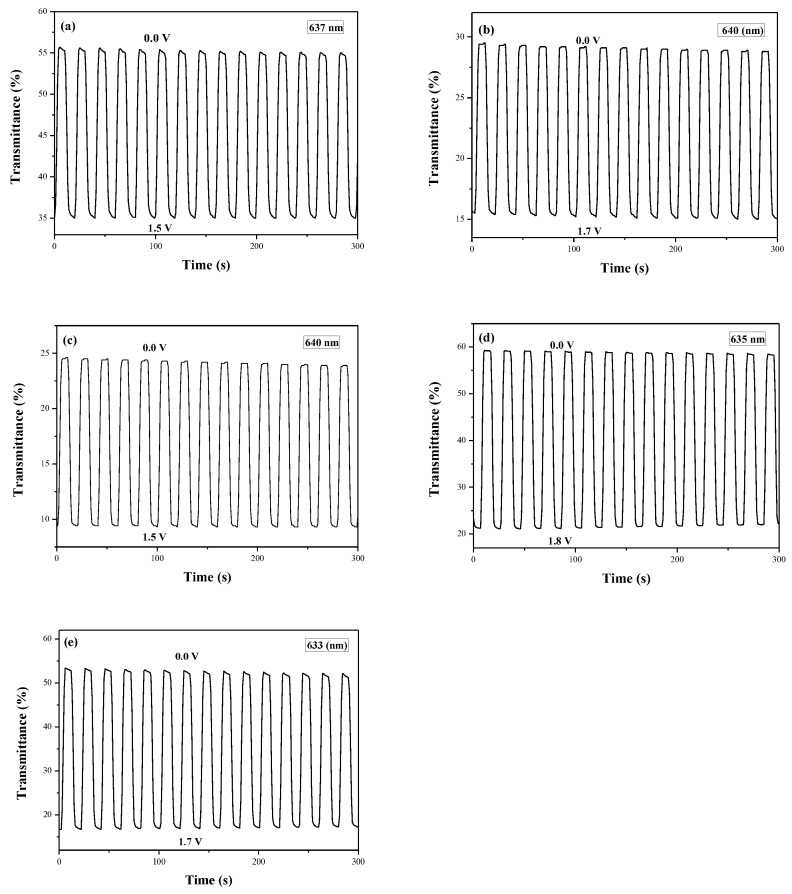
Optical contrast of (**a**) PDiCP/PEDOT-PSS; (**b**) P(DiCP-*co*-CPDT)/PEDOT-PSS; (**c**) P(DiCP-*co*-CPDT2)/PEDOT-PSS; (**d**) P(DiCP-*co*-CPDTK)/PEDOT-PSS; and (**e**) P(DiCP-*co*-CPDTK2)/PEDOT-PSS ECDs with a residence time of 10 s.

Figure 8 showed the electrochromic switching profiles of PDiCP/PEDOT-PSS, P(DiCP-*co*-CPDT)/PEDOT-PSS, P(DiCP-*co*-CPDT2)/PEDOT-PSS, P(DiCP-*co*-CPDTK)/PEDOT-PSS, and P(DiCP-*co*-CPDTK2)/PEDOT-PSS ECDs, the transmittance variations of these ECDs were carried out between 0.0 V and 1.8 V with a time interval of 10 s. The Δ*T*, ΔOD, *Q*_d_, *η*, *τ*_c_, and *τ*_b_ of PDiCP/PEDOT-PSS, P(DiCP-*co*-CPDT)/PEDOT-PSS, P(DiCP-*co*-CPDT2)/PEDOT-PSS, P(DiCP-*co*-CPDTK)/PEDOT-PSS, and P(DiCP-*co*-CPDTK2)/PEDOT-PSS ECDs estimated at the second cycle are listed in Table 6.

P(DiCP-*co*-CPDTK)/PEDOT-PSS and P(DiCP-*co*-CPDTK2)/PEDOT-PSS ECDs revealed larger Δ*T* than that of PDiCP/PEDOT-PSS ECD, whereas P(DiCP-*co*-CPDT)/PEDOT-PSS and P(DiCP-*co*-CPDT2)/PEDOT-PSS ECDs revealed smaller Δ*T* than that of PDiCP/PEDOT-PSS ECD, implying the incorporation of P(DiCP-*co*-CPDTK) and P(DiCP-*co*-CPDTK2) as the anodically-colouring polymers led to a higher Δ*T* than those of PDiCP, P(DiCP-*co*-CPDT), and P(DiCP-*co*-CPDT2) electrodes. In other aspects, the colouration efficiencies of PDiCP/PEDOT-PSS, P(DiCP-*co*-CPDT)/PEDOT-PSS, P(DiCP-*co*-CPDT2)/PEDOT-PSS, P(DiCP-*co*-CPDTK)/PEDOT-PSS, and P(DiCP-*co*-CPDTK2)/PEDOT-PSS ECDs were 259.7 mC cm^−2^ at 637 nm, 358.0 mC cm^−2^ at 640 nm, 461.5 mC cm^−2^ at 640 nm, 633.8 mC cm^−2^ at 635 nm, and 510.4 mC cm^−2^ at 633 nm, respectively. The colouration efficiencies of P(DiCP-*co*-CPDT)/PEDOT-PSS, P(DiCP-*co*-CPDT2)/PEDOT-PSS, P(DiCP-*co*-CPDTK)/PEDOT-PSS, and P(DiCP-*co*-CPDTK2)/PEDOT-PSS ECDs were greater than that of PDiCP/PEDOT-PSS ECD.

The comparisons of transmittance changes and colouration efficiencies with reported ECDs were displayed in Table 7, P(DiCP-*co*-CPDTK)/PEDOT-PSS ECD showed higher transmittance change than those reported for PtCz/PProDOT-Me_2_ [46], poly(2,5-bis(9-methyl-9H-carbazol-3-yl)-1,3,4-oxadiazole)/PEDOT [47], poly(4,4′-di(*N*-carbazolyl)biphenyl)/PEDOT ECDs [48], and was comparable to those reported for poly(4,4′-di(*N*-carbazoyl)biphenyl-*co*-4H-cyclopenta[2,1-b:3,4-b′]dithiophene)/PEDOT [49] and P(tnCz1-*co*-bTp2)/PProDOT-Me_2_ ECDs [50]. On the other side, P(DiCP-*co*-CPDTK)/PEDOT-PSS ECD revealed higher *η* at 635 nm than those reported for PtCz/PProDOT-Me_2_ [46], poly(4,4′-di(*N*-carbazoyl)biphenyl-*co*-4H-cyclopenta[2,1-b:3,4-b′]dithiophene)/PEDOT [49], and P(tnCz1-*co*-bTp2)/PProDOT-Me_2_ ECDs [50], which made P(DiCP-*co*-CPDTK)/PEDOT-PSS ECD desirable for applications in rear-view mirrors of vehicles.

### 3.4. Optical Memory of ECDs

**Figure 9:**
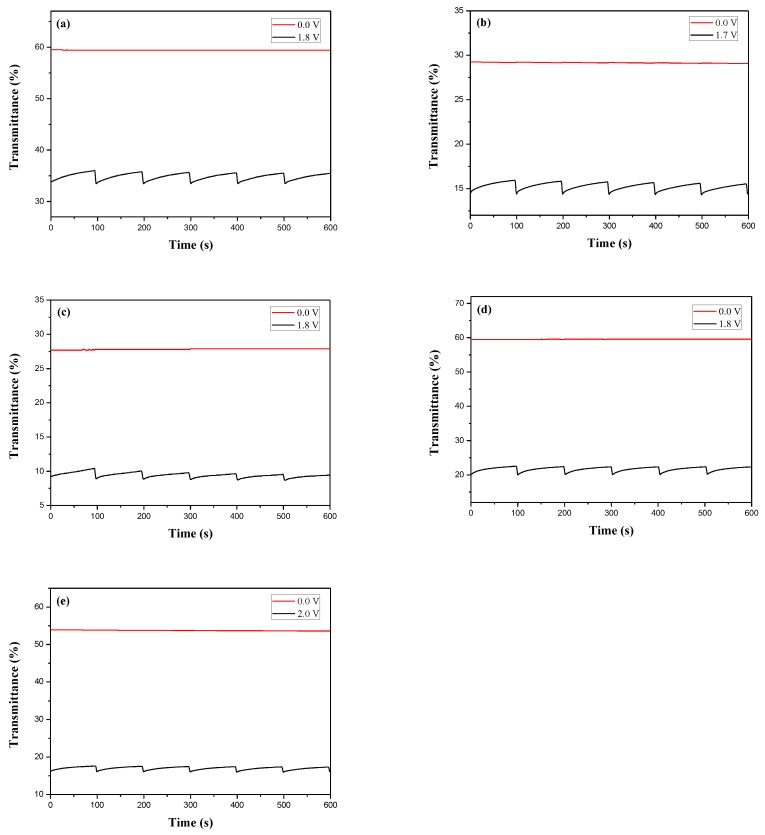
Open circuit stability of (**a**) PDiCP/PEDOT-PSS; (**b**) P(DiCP-*co*-CPDT)/PEDOT-PSS; (**c**) P(DiCP-*co*-CPDT2)/PEDOT-PSS; (**d**) P(DiCP-*co*-CPDTK)/PEDOT-PSS; and (**e**) P(DiCP-*co*-CPDTK2)/PEDOT-PSS devices.

The ability to maintain a bleached (or coloured) state at open circuit of PDiCP/PEDOT-PSS, P(DiCP-*co*-CPDT)/PEDOT-PSS, P(DiCP-*co*-CPDT2)/PEDOT-PSS, P(DiCP-*co*-CPDTK)/PEDOT-PSS, and P(DiCP-*co*-CPDTK2)/PEDOT-PSS ECDs was measured in bleached and coloured states by applying a potential for 1 s in a time interval of each 100 s [51]. As shown in Figure 9, the PDiCP/PEDOT-PSS, P(DiCP-*co*-CPDT)/PEDOT-PSS, P(DiCP-*co*-CPDT2)/PEDOT-PSS, P(DiCP-*co*-CPDTK)/PEDOT-PSS, and P(DiCP-*co*-CPDTK2)/PEDOT-PSS ECDs showed almost no transmittance change in bleached state, displaying satisfactory optical memories in bleached state. However, ECDs were rather less stable than those in the bleached state, but the transmittance variations were less than 4% in the coloured state, indicating the PDiCP/PEDOT-PSS, P(DiCP-*co*-CPDT)/PEDOT-PSS, P(DiCP-*co*-CPDT2)/PEDOT-PSS, P(DiCP-*co*-CPDTK)/PEDOT-PSS, and P(DiCP-*co*-CPDTK2)/PEDOT-PSS ECDs had desirable optical memories.

### 3.5. Redox Stability of ECDs

**Figure 10:**
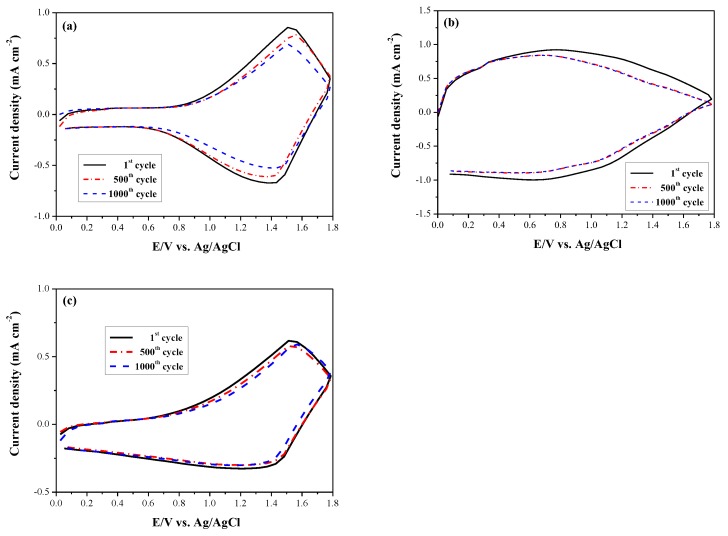
Cyclic voltammograms of (**a**) PDiCP/PEDOT-PSS; (**b**) P(DiCP-*co*-CPDT2)/PEDOT-PSS; and (**c**) P(DiCP-*co*-CPDTK)/PEDOT-PSS devices with a scan rate of 500 mV s^−1^ at the first, 500^th^, and 1000^th^ cycles.

The multiple switching stability of PDiCP/PEDOT-PSS, P(DiCP-*co*-CPDT2)/PEDOT-PSS, and P(DiCP-*co*-CPDTK)/PEDOT-PSS ECDs was monitored using CV at potentials between 0.0 and 1.8 V with a scan rate of 500 mV s^−^^1^ as displayed in Figure 10 [52]. Electrochemical activity of 89.0%, 87.2%, and 92.4% was preserved after 500 cycles, respectively, and 81.2%, 86.6%, and 88.8%, respectively, of electrochemical activity was preserved after 1000 cycles for PDiCP/PEDOT-PSS, P(DiCP-*co*-CPDT2)/PEDOT-PSS, and P(DiCP-*co*-CPDTK)/PEDOT-PSS ECDs. P(DiCP-*co*-CPDT2)/PEDOT-PSS and P(DiCP-*co*-CPDTK)/PEDOT-PSS ECDs employed copolymers as anodic electrodes presented better multiple cycling stability than that of PDiCP (homopolymer)/PEDOT-PSS ECD. Considering the above consequences, these ECDs displayed adequate redox stability for electrochromic applications.

## 4. Conclusions

An anodic homopolymer (PDiCP) and four copolymers (P(DiCP-*co*-CPDT), P(DiCP-*co*-CPDT2), P(DiCP-*co*-CPDTK), and P(DiCP-*co*-CPDTK2)) were prepared electrochemically. PDiCP homopolymer film was light grey in the neutral state, dark khaki in the intermediate state, and grey black in the oxidized states. For the copolymer films, P(DiCP-*co*-CPDT) and P(DiCP-*co*-CPDT2) films were light brown at 0.0 V, light cadet blue at 0.4 V, and navy blue at 1.2 (or 1.3 V), whereas P(DiCP-*co*-CPDTK) and P(DiCP-*co*-CPDTK2) films were light yellow at 0.0 V, grey at 0.8 V, and rock grey at 1.1 V. Electrochromic switching characterizations of anodically-colouring polymers revealed high Δ*T* and *η* for P(DiCP-*co*-CPDTK) film, which were determined as 39.5% and 184.1 cm^2^ C^−1^ at 1037 nm, respectively. Five dual-type ECDs based on PDiCP, P(DiCP-*co*-CPDT), P(DiCP-*co*-CPDT2), P(DiCP-*co*-CPDTK), and P(DiCP-*co*-CPDTK2) films as anodically-colouring polymers and PEDOT-PSS film as cathodically-colouring polymer were fabricated and their spectroelectrochemical properties were characterized. P(DiCP-*co*-CPDTK)/PEDOT-PSS ECD revealed light silverish-yellow, light grey, grey, light greyish blue, and greyish blue at 0.0 V, 0.7 V, 1.3 V, 1.7 V, and 2.0 V, respectively. P(DiCP-*co*-CPDTK)/PEDOT-PSS and P(DiCP-*co*-CPDTK2)/PEDOT-PSS ECDs showed high Δ*T* (38.2% and 36.0% for P(DiCP-*co*-CPDTK)/PEDOT-PSS and P(DiCP-*co*-CPDTK2)/PEDOT-PSS ECDs, respectively), high *η* (633.8 and 510.4 mC cm^−2^ for P(DiCP-*co*-CPDTK)/PEDOT-PSS and P(DiCP-*co*-CPDTK2)/PEDOT-PSS ECDs, respectively), and high ΔOD values. In addition, P(DiCP-*co*-CPDTK)/PEDOT-PSS displayed suitable redox stability and optical memory.

## Figures and Tables

**Table 1:** Feed species of anodically-colouring polymers (a)–(e).

Electrodes	Anodically Colouring Polymers	Feed Species of Anodic Polymer	Feed Molar Ratio of Anodic Polymer
(a)	PDiCP	4 mM DiCP	DiCP
(b)	P(DiCP-*co*-CPDT)	2 mM DiCP + 2 mM CPDT	DiCP:CPDT = 1:1
(c)	P(DiCP-*co*-CPDT2)	2 mM DiCP + 4 mM CPDT	DiCP:CPDT = 1:2
(d)	P(DiCP-*co*-CPDTK)	2 mM DiCP + 2 mM CPDTK	DiCP:CPDTK = 1:1
(e)	P(DiCP-*co*-CPDTK2)	2 mM DiCP + 4 mM CPDTK	DiCP:CPDTK = 1:2

**Table 2:** Colorimetric values (L*, a*, and b*), CIE chromaticity values (*x*, *y*), and CIE diagrams of (a) PDiCP, (b) P(DiCP-*co*-CPDT2), and (c) P(DiCP-*co*-CPDTK) films at various applied potentials.

Films	Potential (V)	L*	a*	b*	*x*	*y*	Diagrams
PDiCP	0.0	95.00	0.27	1.75	0.44	0.40	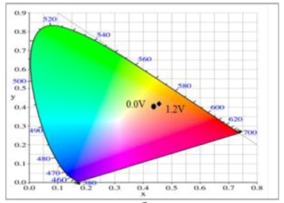
	0.5	94.98	0.30	1.76	0.44	0.40	
	0.7	95.00	0.26	1.86	0.44	0.40	
	0.9	94.60	0.09	2.32	0.45	0.40	
	1.0	94.23	0.00	4.47	0.45	0.41	
	1.2	93.01	−0.43	11.73	0.45	0.41	
DiCP-*co*-CPDT2)	0.0	33.57	11.70	23.10	0.53	0.41	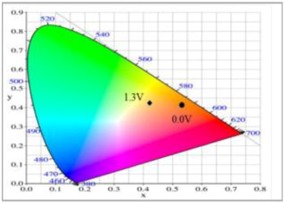
	0.6	38.97	−1.73	12.83	0.46	0.43	
	0.7	36.72	−3.15	5.23	0.44	0.42	
	0.9	35.03	−3.84	−3.05	0.42	0.41	
	1.1	34.60	−4.40	−3.72	0.42	0.41	
	1.3	34.66	−5.80	−0.16	0.42	0.42	
P(DiCP-*co*-CPDTK)	0.0	86.69	−0.54	5.95	0.45	0.41	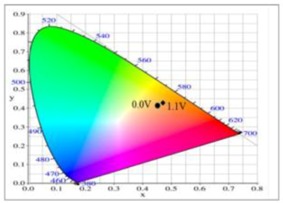
	0.4	85.69	−0.26	6.82	0.45	0.41	
	0.7	85.11	0.15	7.04	0.45	0.41	
	0.8	84.77	0.40	8.94	0.45	0.41	
	0.9	84.47	0.45	12.53	0.46	0.41	
	1.1	84.48	1.93	14.06	0.46	0.42	

**Table 3:** Optical and electrochemical properties investigated at the selected applied wavelength for the electrodes.

Electrodes	λ (nm)	*T*_ox_	*T*_red_	Δ*T*	ΔOD	Q*_d_* (mC cm^−2^)	*η* (cm^2^ C^−1^)	*τ*_c_ (s)	*τ*_b_ (s)
PDiCP	1025	53.1	71.9	18.8	0.13	1.05	123.8	1.9	4.5
P(DiCP-*co*-CPDT)	1034	12.3	40.6	28.3	0.51	3.41	149.5	4.0	3.5
P(DiCP-*co*-CPDT2)	1034	4.6	33.7	29.1	0.86	10.63	80.9	6.7	3.9
P(DiCP-*co*-CPDTK)	1037	17.1	56.6	39.5	0.51	2.77	184.1	2.7	3.2
P(DiCP-*co*-CPDTK2)	890	38.2	74.1	35.9	0.28	2.51	111.5	1.9	2.4

**Table 4:** Transmittance changes and colouration efficiencies of carbazole-based polymer films.

Polymer Films	Δ*T*_max_ (%)	*η* (cm^2^ C^−1^)	Ref.
PETCB	36 (1100 nm)	---	41
PMCzP	29 (460 nm)	---	42
PBCz	18.6 (1050 nm)	180.3	43
P(BCz1-*co*-Inc1)	43.0 (787 nm)	148	44
P(NO_2_-3Cz)	52 (710 nm)	35	45
P(DiCP-*co*-CPDTK)	39.5 (1037 nm)	184.1	This work

**Table 5:** Electrochromic photographs, colourimetric values (L*, a*, and b*), CIE chromaticity values (*x*, *y*), and CIE diagram of P(DiCP-*co*-CPDTK)/PEDOT-PSS ECD at various applied potentials.

Potential (V)	Photographs	L*	a*	b*	*x*	*y*	Diagram
0.0	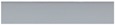	81.81	−3.79	−0.90	0.43	0.41	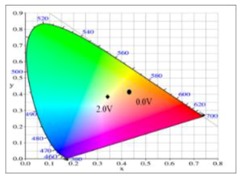
0.7	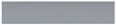	79.79	−3.16	−1.02	0.44	0.41	
1.3	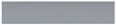	68.79	−10.12	−10.64	0.41	0.40	
1.7	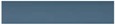	61.27	−16.09	−21.18	0.37	0.39	
2.0	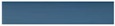	55.36	−19.91	−29.70	0.34	0.38	

**Table 6:** Optical and electrochemical properties investigated at the selected applied wavelength for the devices.

Devices	λ (nm)	*T*_ox_	*T*_red_	Δ*T*	ΔOD	Q*_d_* (mC cm^−2^)	*η* (cm^2^ C^−1^)	τ_c_ (s)	τ_b_ (s)
PDiCP/PEDOT-PSS	637	35.1	55.6	20.5	0.20	0.70	259.7	2.7	2.0
P(DiCP-*co*-CPDT)/PEDOT-PSS	640	15.3	29.5	14.2	0.29	0.81	358.0	2.5	2.1
P(DiCP-*co*-CPDT2)/PEDOT-PSS	640	9.4	24.7	15.3	0.42	0.91	461.5	2.6	2.7
P(DiCP-*co*-CPDTK)/PEDOT-PSS	635	21.1	59.3	38.2	0.45	0.71	633.8	2.4	2.5
P(DiCP-*co*-CPDTK2)/PEDOT-PSS	633	17.1	53.1	36.0	0.49	0.96	510.4	2.6	2.4

**Table 7:** Transmittance changes and colouration efficiencies of ECDs.

ECD Configuration	Δ*T*_max_ (%)	*η*_max_ (cm^2^ C^−1^)	Ref.
PtCz/PProDOT-Me_2_	36 (572 nm)	343.4 (572 nm)	46
poly(2,5-bis(9-methyl-9H-carbazol-3-yl)-1,3,4-oxadiazole)/PEDOT	35 (620 nm)	---	47
poly(4,4′-di(*N*-carbazolyl)biphenyl)/PEDOT	19 (550 nm)	---	48
poly(4,4′-di(*N*-carbazoyl)biphenyl-*co*- 4H-cyclopenta[2,1-b:3,4-b′]dithiophene)/PEDOT	39.8 (628 nm)	319.98 (628 nm)	49
P(tnCz1-*co*-bTp2)/PProDOT-Me_2_	40 (630 nm)	519 (630 nm)	50
P(DiCP-*co*-CPDTK)/PEDOT-PSS	38.2 (635 nm)	634 (635 nm)	This work
